# The *(R)*-enantiomer of the 6-chromanol derivate SUL-121 improves renal graft perfusion via antagonism of the α_1_-adrenoceptor

**DOI:** 10.1038/s41598-018-36788-0

**Published:** 2019-01-09

**Authors:** D. Nakladal, H. Buikema, A. Reyes Romero, S. P. H. Lambooy, J. Bouma, G. Krenning, P. Vogelaar, A. C. van der Graaf, M. R. Groves, J. Kyselovic, R. H. Henning, L. E. Deelman

**Affiliations:** 1 Department of Clinical Pharmacy and Pharmacology, University of Groningen, University Medical Center Groningen, Hanzeplein 1, 9713GZ Groningen, The Netherlands; 2 Department of Pharmacology & Toxicology, Faculty of Pharmacy, Comenius University in Bratislava, Odbojárov 10, 832 32 Bratislava, Slovakia; 3 Department of Drug Design, School of Pharmacy, University of Groningen, Antonius Deusinglaan 1 Postbus 196, 9700 AD Groningen, The Netherlands; 4 Cardiovascular Regenerative Medicine, Dept. Pathology and Medical Biology, University of Groningen, University Medical Center Groningen, Groningen, The Netherlands; 5 Sulfateq B.V., Admiraal de Ruyterlaan 5, 9726GN Groningen, The Netherlands; 6 5th Department of Internal Medicine, Faculty of Medicine, University Hospital, Commenius University, Bratislava, Slovak Republic

## Abstract

SUL-compounds are protectants from cold-induced ischemia and mitochondrial dysfunction. We discovered that adding SUL-121 to renal grafts during warm machine reperfusion elicits a rapid improvement in perfusion parameters. Therefore, we investigate the molecular mechanisms of action in porcine intrarenal arteries (PIRA). Porcine kidneys were stored on ice overnight and perfusion parameters were recorded during treatment with SUL-compounds. Agonist-induced vasoconstriction was measured in isolated PIRA after pre-incubation with SUL-compounds. Receptor binding and calcium transients were assessed in α_1_-adrenoceptor (α_1_-AR) transgenic CHO cells. Molecular docking simulation was performed using Schrödinger software. Renal pressure during warm reperfusion was reduced by SUL-121 (−11.9 ± 2.50 mmHg) and its *(R)-*enantiomer SUL-150 (−13.2 ± 2.77 mmHg), but not by the *(S)-*enantiomer SUL-151 (−1.33 ± 1.26 mmHg). Additionally, SUL-150 improved renal flow (16.21 ± 1.71 mL/min to 21.94 ± 1.38 mL/min). SUL-121 and SUL-150 competitively inhibited PIRA contraction responses to phenylephrine, while other 6-chromanols were without effect. SUL-150 similarly inhibited phenylephrine-induced calcium influx and effectively displaced [7-Methoxy-^3^H]-prazosin in CHO cells. Docking simulation to the α_1_-AR revealed shared binding characteristics between prazosin and SUL-150. SUL-150 is a novel α_1_-AR antagonist with the potential to improve renal graft perfusion after hypothermic storage. In combination with previously reported protective effects, SUL-150 emerges as a novel protectant in organ transplantation.

## Introduction

Although living donor transplantation may lead to superior recipient outcomes, 67% of all renal transplantations in the Netherlands depend on donations after circulatory death (DCD) or donations after brain death (DBD)^1^. Unfortunately, up to 8% of the DCD and DBD kidneys obtained for donation are eventually not used for transplantation. Although the reasons for discarding offered transplant kidneys may be diverse, a study performed in the UK indicates that poor renal perfusion with preservation fluid after procurement is a common reason^2^. In addition to perfusion problems encountered at the time of procurement, vascular changes are also observed during hypothermic storage of the donated kidneys^3^. These changes may include damage to the endothelium, resulting in the impaired production and release of vasodilatory components, ultimately leading to increased renal resistance. The importance of maintaining renal perfusion may be further illustrated by a study demonstrating that machine-measured renal resistance is a significant predictor of 1-year allograft outcome^4^.

In recent years, our group has developed a new class of compounds derived from 6-chromanol (SUL compounds) to aid in the hypothermic preservation of tissue and cells. SUL-109 was initially described as a preservation agent that protects from damage associated with hypothermia and reactive oxygen species (ROS) in adipose-derived stem cells^5^. Specifically, SUL-109 preserved the mitochondrial network structure and activated mitochondrial complexes I and IV, thereby preventing ROS formation while maintaining ATP production.

Recently, we demonstrated the effectiveness of SUL compounds against hypothermic damage in the kidney as SUL-109 and the related SUL-121 compound ameliorated renal injury after deep hypothermia and rewarming in rats by preserving mitochondrial mass, function and ATP levels^6^. Moreover, the protective effects of SUL compounds have now been demonstrated in several disease models characterized by mitochondrial dysfunction. In type 2 diabetes, treatment with SUL-121 halted the progression of kidney damage and prevented endothelial dysfunction through a mechanism involving lowered ROS production^7^. Further, in chronic obstructive pulmonary disease, SUL-121 also reduced ROS production, resulting in reduced LPS-induced airway hyper reactivity and neutrophil influx and increased hydrogen sulfide (H_2_S) production^8^.

Although we thoroughly investigated the underlying protective mechanisms of SUL-109 and SUL-121 on the kidney during hypothermia, some observations were left unaddressed. Interestingly, SUL-121 caused a significant further reduction in blood pressure during the hypothermic phase compared to controls not receiving SUL-121^6^. In addition, we now report that SUL-121 induced a profound and immediate increase in renal flow during warm machine reperfusion of cold-stored porcine kidneys. To further explore these putative vascular effects of SUL-121, we here investigate the effects of SUL-121 and its enantiomers on renal machine reperfusion and on vasomotor function of porcine intra-renal arterial segments.

## Results

### Effects of SUL-121 and its enantiomers SUL-150 and SUL-151 on renal perfusion pressure and flow

**Figure 1:**
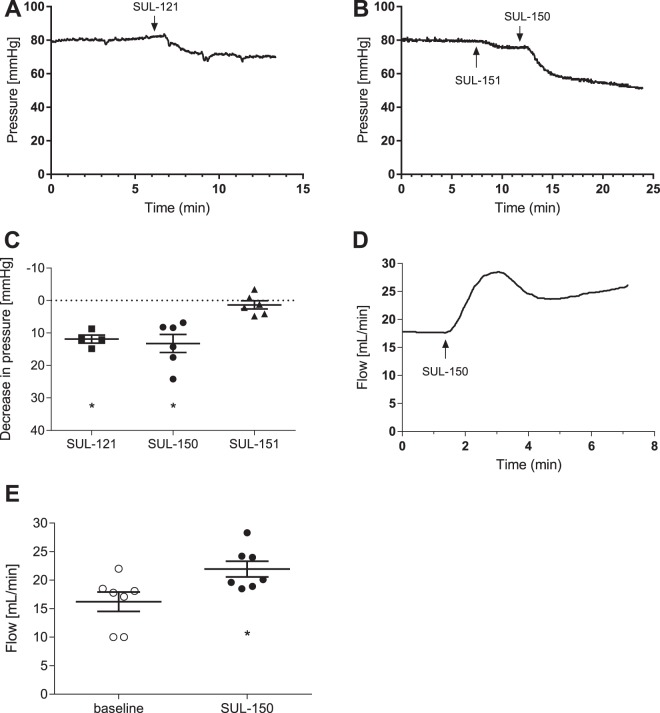
Effect of SUL-compounds on renal perfusion pressure and flow during machine reperfusion of cold-stored porcine kidneys. (**A**) Typical recording of the effect of SUL-121 (50 µM) on perfusion pressure. (**B**) Typical recording of the effects of SUL-151 (*(S)*-enantiomer of SUL-121, 50 µM) and SUL-150 (*(R)*-enantiomer of SUL-121, 50 µM) on perfusion pressure. (**C**) Average decrease in perfusion pressure by SUL-121 (n = 4), SUL-150 (n = 6) and SUL-151 (n = 6) *P < 0.05 vs baseline pressure. (**D**) Typical recording of the effect of SUL-150 (50 µM) on renal perfusion flow. (**E**) Average increase in perfusion flow by SUL-150 (50 µM, n = 7) *P < 0.05 vs baseline flow.

Cold-stored porcine kidneys were rewarmed to 20 °C with warm oxygenated medium while flow-rate was kept constant and occasionally adjusted to maintain a pressure of 80 mmHg (data not shown). Addition of SUL-121 to the circulating medium caused a profound decrease in renal pressure (Fig. 1A,C; −11.9 ± 2.5 mmHg). SUL-151, the *(S)*-enantiomer of SUL-121 demonstrated only a minor effect on renal pressure (Fig. 1B,C; −1.3 ± 1.3 mmHg), whereas a subsequent addition of SUL-150, the *(R)*-enantiomer of SUL-121, caused a decrease in pressure similar to SUL-121 (Fig. 1B,C; −13.2 ± 2.8 mmHg). These experiments therefore demonstrate that the effects of SUL-121 on renal perfusion pressure are mediated predominantly through the *(R)*-enantiomer (SUL-150).

To investigate how the observed decrease in renal resistance by SUL-150 would benefit renal perfusion flow, additional experiments were performed with fixed pressure (80 mmHg) and dynamic flow adjustment. SUL-150 caused an approximate 35% increase in renal flow from baseline (Fig. 1D,E; baseline: 16.2 ± 1.7 mL/min, SUL-150: 22.0 ± 1.4 mL/min, p < 0.05).

### Effects of SUL-150 on constriction of isolated porcine intrarenal arteries

**Figure 2:**
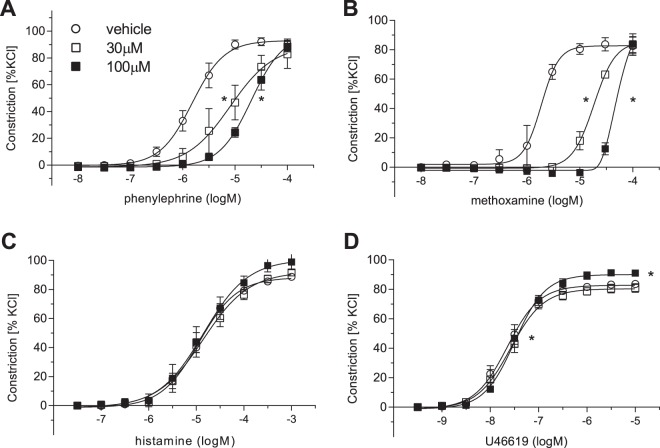
The inhibitory effects of SUL-150 are specific for α_1_-AR. (**A**) SUL-150 dose-dependently inhibited phenylephrine and (**B**) methoxamine-induced vasoconstrictions in isolated intrarenal artery rings. (**C**) SUL-150 did not affect contractions induced by histamine. (**D**) Effect of SUL-150 on U46619 induced contractions. Data from 2–3 experiments (n = 4–6 per group).

To further investigate the putative vasodilatory effects of SUL-150, we explored the effects of pre-incubation with SUL-150 on vasoconstriction of isolated intrarenal arteries to various agonists (Fig. 2). For this, the effects of SUL-150 (30 µM and 100 µM) on cumulative concentration response curves to α_1_-adrenoceptor (α_1_-AR) agonists PE and methoxamine (Fig. 2A,B, respectively), histamine (Fig. 2C) and U46619 (a synthetic thromboxane A2 (TP) receptor agonist, Fig. 2D) were determined. SUL-150 caused a concentration dependent competitive inhibition of the concentration response curves to PE and methoxamine (Fig. 2A,B). SUL-150 did not affect responses to histamine and induced a small, albeit statistically significant shift in EC_50_ and E_max_ of U46619-induced vasoconstriction (Supplementary Tables 1 and 2). Further, these effects are not mediated through the endothelium as removal of the endothelium did not abrogate the effects of SUL-150 on methoxamine induced vasoconstriction (Supplementary Fig. 1A). Rather, our findings suggest a competitive antagonistic effect of SUL-150 that is specific for α_1_-AR. We previously reported that vascular constriction via α_1_-ARs is partly dependent on transactivation of EGF receptors^9,10^. To exclude that inhibition of vasoconstriction by SUL-150 is mediated through altered epidermal growth factor receptor (EGFR) transactivation, additional experiments were performed in isolated blood vessels and cell culture (Supplemental Fig. 2). These experiments demonstrated that SUL-150 antagonism towards α_1_-ARs is indeed not mediated through EGFR transactivation.

### Schild analysis of SUL-121, SUL-150 and SUL-151 antagonism in α_1_-AR mediated vasoconstriction

**Figure 3:**
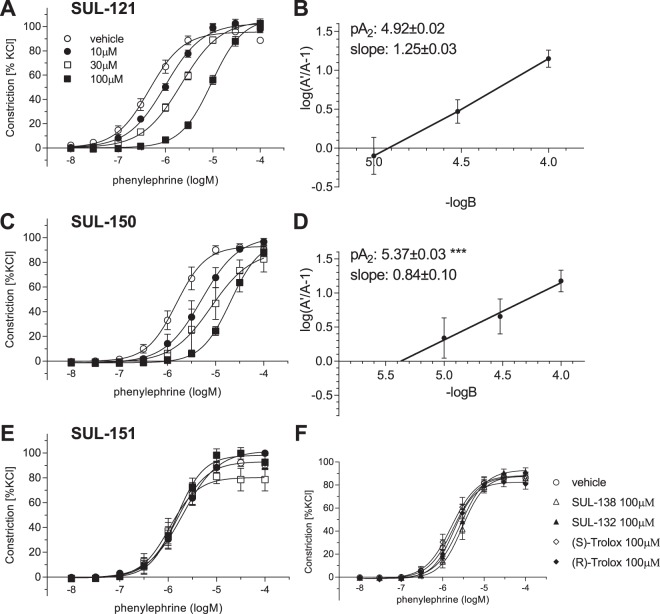
SUL-150, the *(R)-*enantiomer of SUL-121 inhibits phenylephrine mediated contractions in isolated porcine intrarenal arteries. Contractions to phenylephrine were studied in the presence of 10 µM, 30 µM and 100 µM of SUL-121 (**A**) and its enantiomers SUL-150 (**C**) and SUL-151 (**E**). Schild plots for SUL-121 and SUL-150 are shown in panel (**B**) and (**D**), respectively. ***p < 0.001 vs SUL-121. (**F**) Phenylephrine concentration-response curves in PIRA after incubation with molecular structural analogues of SUL-121. Data from 2–3 experiments (n = 4–6 per group).

To further characterize the effects of SUL-121 and its enantiomers on α_1_-AR mediated vasoconstriction, Schild analysis was performed. Concentration response curves to PE in the presence of 10 µM, 30 µM and 100 µM SUL-121, SUL-150 or SUL-151 are shown in Fig. 3. SUL-121 and SUL-150 caused a dose-dependent right shift of the curves with significant changes in EC_50_ (Table 1). Schild comparison of SUL-121 and SUL-150 constriction response curves revealed a lower potency of SUL-121 (pA_2_ = 4.92 ± 0.02) over SUL-150 (pA_2_ = 5.37 ± 0.03), with Schild slopes not deviating significantly from unity (Fig. 3B,D, respectively). Pre-treatment with SUL-151 was without effect and therefore our findings are in line with the results obtained from isolated perfused kidneys.

**Table 1:** Effects of SUL-121, SUL-150 and SUL-151 on pEC_50_ values for PE mediated constrictions in porcine intrarenal arteries.

Compound	Vehicle	Concentration		
		10 µM	30 µM	100 µM
SUL-121	6.34 ± 0.04	6.00 ± 0.04*	5.67 ± 0.04*	5.03 ± 0.05*
SUL-150	5.82 ± 0.05	5.29 ± 0.08*	5.09 ± 0.18*	4.69 ± 0.06*
SUL-151	5.82 ± 0.07	5.69 ± 0.09	5.97 ± 0.08	5.82 ± 0.05

pEC_50_ values are –log transformed. Each value represents the mean ± standard error of the mean (SEM). (n = 4–6 per group). *P < 0.05 vs vehicle.

### Effects of structural analogues of SUL-121 on α_1_-AR mediated vasoconstriction

Next, we screened the effects of additional SUL-121 analogues and/or metabolites on PE-induced constriction of porcine intrarenal artery (PIRA) (Fig. 3F) in order to elucidate the role of individual molecular structural components in the inhibition of α_1_-ARs. Tested were enantiomers of SUL-109 (*(S)-*enantiomer SUL-138 and *(R)-*enantiomer SUL-132) which contain a terminal -N-(2-hydroxyethyl) group, and Trolox enantiomers which are products of -piperazine-1-carboxamide hydrolysis of SUL-121^5^. The enantiomers of Trolox did not influence α_1_-AR mediated constriction of porcine intrarenal arteries, suggesting importance of -piperazine-1-carboxamide in binding to α_1_-ARs. Additional substitution of piperazine-1-carboxamide N-terminal of SUL-150 with -(2-hydroxyethyl) yielding *(R)*-SUL-109 caused a loss of α_1_-AR antagonistic effect.

### Effects of SUL-150 on α_1_-AR subtypes

**Figure 4:**
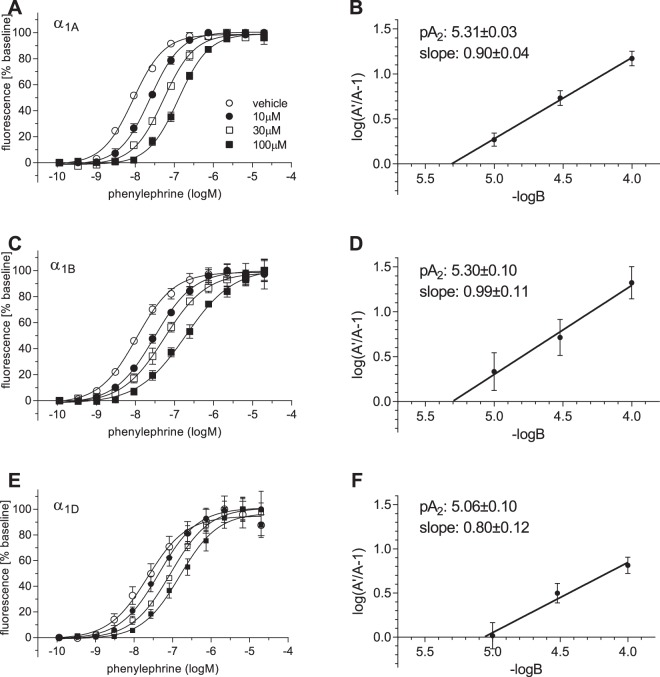
SUL-150 inhibits intracellular signalling mediated through α_1_-AR. Intracellular calcium measurements in transgenic CHO cells overexpressing α_1_-AR subtypes 1A (**A**),  1B (**C**) and 1D (**E**) after stimulation with phenylephrine in the presence of 10 µM, 30 µM and 100 µM SUL-150. Respective Schild plots are shown in panels (**B**), (**D**) and (**F**). Data from 2–3 experiments (n = 4–6 per group).

To further investigate the specificity of SUL-150 for inhibition of α_1_-AR mediated vasoconstriction, PE-induced calcium transients were studied in CHO cells stably overexpressing the human α_1_-AR subtypes A, B and D. SUL-150 shifted dose response curves rightwards for all three α_1_-AR isoforms (Fig. 4A,C and E, Table 2). Schild analysis for the three α_1_-AR subtypes is shown in Fig. 4B,D and F. For α_1_-AR subtypes A and B, pA_2_ values were similar (5.31 ± 0.03 and 5.30 ± 0.10 respectively) to the pA_2_ obtained from the inhibitory effects of SUL-150 on vascular contraction (5.37 ± 0.03). A significantly lower pA_2_ was found for α_1_-AR subtype D (5.06 ± 0.10, p < 0.05). SUL-151 did not affect calcium transients in α_1_-AR transfected CHO cells (Supplementary Fig. 1B). In line with our data from vascular measurements, SUL-150 did not have profound effects on U46619 and histamine induced calcium transients in Hela cells (Supplementary Fig. 1C,D).

**Table 2:** Effects of SUL-150 on pEC_50_ values for PE mediated calcium signaling in CHO cells stably transformed with adrenoceptor α_1A_, α_1B_ or α_1D_.

Cell line	vehicle	Concentration of SUL-150		
		10 µM	30 µM	100 µM
CHO + α_1A_	8.07 ± 0.03	7.62 ± 0.03*	7.28 ± 0.03*	6.88 ± 0.03*
CHO + α_1B_	7.99 ± 0.08	7.51 ± 0.08*	7.24 ± 0.07*	6.72 ± 0.08*
CHO + α_1D_	7.68 ± 0.13	7.37 ± 0.10	7.04 ± 0.06*	6.80 ± 0.10*

pEC_50_ values are –log transformed. Each value represents the mean ± standard error of the mean (SEM). (n = 4–6 per group). *P < 0.05 vs vehicle.

### Radioligand binding assay in α_1A_-AR overexpressing CHO cells

**Figure 5:**
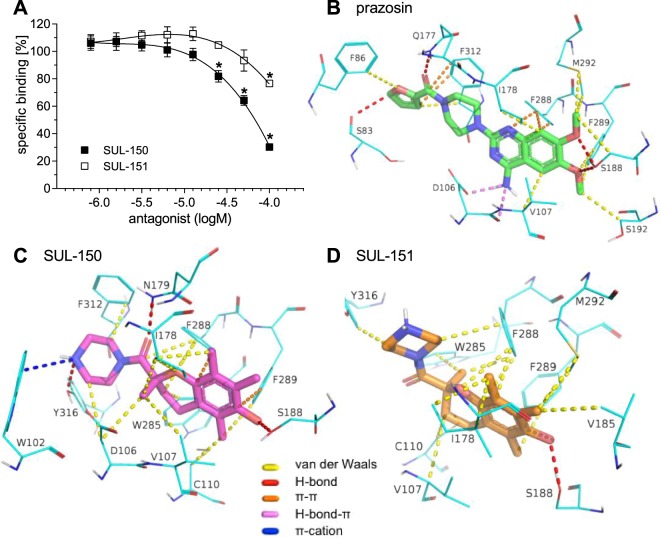
SUL-150 displaces prazosin from the α_1A_-AR. Displacement of tritium labelled prazosin by SUL-150 and SUL-151 in α_1A_-AR transgenic CHO cells. *p < 0.05 vs baseline, n = 4 per concentration in each group. Competition mode of action of prazosin and SUL-compounds based on Induced-fit molecular docking simulation. Polar 6,7-methoxy groups of prazosin and 6-hydroxy of SUL-compounds are oriented towards TM5 and the polar surface of the antagonist binding pocket. The corresponding binding modes for prazosin (**B**), SUL-150 (**C**) and SUL-151 (**D**) are shown below with α_1_-AR residues depicted in cyan.

To explore whether SUL-150 directly interacts with the α_1_-AR as a receptor antagonist, a competitive binding assay was performed on α_1A_-AR transgenic CHO cells using radiolabelled prazosin, an established α_1A_-AR antagonist. Incubation with SUL-150 induced a significant displacement of the radioligand starting at 25 µM concentrations indicating higher potency than SUL-151, which demonstrated significant displacement only at the highest concentration (100 µM, Fig. 5A).

### Induced fit molecular docking simulation

To explain experimentally determined differences between SUL-150 and SUL-151 in competitive binding to the antagonist site on the α_1A_-AR, Induced Fit molecular docking simulation was performed using prazosin as a reference. As no crystal structure is yet available for α_1_-AR a homology model was built using SWISS-MODEL. Manual evaluation of the resulting models identified an optimal homology between α_1_-AR and D3 dopamine receptor, with 36% sequence identity and 68% coverage. Validation using Ramachandran plot suggested 0.96% of residues as outliers, specifically PRO242, ASP228 and PRO299. In addition, 3.21% of residues were in allowed regions and 95.83% in the preferred region.

Prazosin coexists in two protonation forms at pH 7.4. In the protonated form, the N1 assumes a positive charge, subsequently forming a salt bridge with the negatively charged side chain of ASP106, ultimately causing this form to assume an inverted orientation relative to its non-protonated form (Fig. 5B). The quinazoline scaffold of non-protonated prazosin was docked close to TM5 to form a hydrogen bond between the 6,7-methoxy groups and SER188^11,12^, a hydrogen bond between the furan oxygen and SER83, and between the prazosin carboxamide and GLN177 side chain; van der Waals interactions with side chains of PHE86, VAL107, ILE178, SER192^11^, PHE289^13^, MET292 and PHE312^12,14^; π-π interactions with PHE288 and PHE312^12^; and a π-hydrogen bond interaction with the side chain carboxyl group and backbone peptide carboxamide of ASP106^12,15^. Thus, the proposed binding mode of non-protonated prazosin utilized interactions that were in accord with those described in previous literature.

Following the generation of a reference prazosin binding site, an induced fit of SUL-compounds was performed (Fig. 5C,D). Both SUL-150 and SUL-151 demonstrated alignment of the chromane scaffold with prazosin quinazoline, 6-hydroxy groups (SUL) and 6-metoxy (prazosin) as well as over their common piperazine moiety. Residues which were involved in forming contacts with all three compounds were VAL107, ILE178, SER188, PHE288, PHE289. Glide scores computed using the Schrödinger Small-Drug Discovery Suite were −10.8 kcal × mol^−1^, −10.2 kcal × mol^−1^ and −9.4 kcal × mol^−1^ for prazosin, SUL-150 and SUL-151, respectively, which corresponded to a difference in potency (prazosin pA_2_ for PE 8.93 ± 0.04^16^, SUL-150 5.37 ± 0.03). Interestingly, prazosin and SUL-150 (Fig. 5C) formed contacts with PHE312 and ASP106 confirmed in prazosin binding by mutagenesis^11,15^, whereas SUL-151 (Fig. 5D) did not show interactions with these residues. Additionally, the chirality of SUL-150 enables the orientation of its carboxamide towards ASN179, effectively forming a hydrogen bond. Furthermore, the protonated N-terminal of SUL-150 formed an additional hydrogen bond with TYR316 and a π-cation interaction with TRP102, known to frequently appear among aromatic amino acids^17^. Taken together, our model suggests that SUL-150 has a similar α_1_-AR binding profile to prazosin, considering the orientation of core ligand scaffolds in the antagonist binding pocket and formation of contacts with key receptor residues.

## Discussion

Previously, we demonstrated that 6-chromanol derived SUL-compounds are protective in models of type 2 diabetes^7^, long-term hypothermic storage^5^ and chronic obstructive pulmonary disease^8^. Further, the molecular mechanisms of SUL-compounds were studied in detail in adipose-tissue-derived stem cells demonstrating that compound SUL-109 activated mitochondrial membrane complexes I and IV thereby alleviating mitochondrial ROS production and preserving ATP production^5^. In addition to the effects of SUL-compounds on the mitochondrial respiratory chain, we now demonstrate that SUL-121, particularly through its *(R)-*enantiomer SUL-150, has antagonistic effects on α_1_-AR mediated vasoconstriction in isolated porcine kidneys.

The present finding that SUL-150 improved renal machine perfusion parameters through antagonism of α_1_-AR, indicates active α_1_-AR signalling during rewarming of cold-stored kidneys. At present, only limited data is available on the role of α_1_-AR in renal machine perfusion. However, it was reported that treatment of living donors with the α_1_-AR antagonist phentolamine improved flow and decreased resistance of machine-perfused graft kidneys, and promoted early allograft function after transplantation^18^. In a separate study, inhibition of the Rho-kinase pathway in porcine kidneys during cold storage resulted in higher flow upon reperfusion, as well as improved glomerular function and tubular cell integrity^19^. Considering the involvement of α_1_-ARs in Rho-kinase signalling, these results may further highlight the importance of vascular α_1_-AR in the regulation of kidney perfusion. In addition, in an experimental model of donation after brain death, massive sympathetic discharge as a result of brain death was shown to induce hemodynamic injury, resulting in increased ischemia reperfusion to the transplanted lung^20^. Interestingly, these effects could be blocked by treatment with the α_1_-AR antagonist prazosin. Taken together, these findings support the importance of α_1_-AR as a target to treat vascular injury in transplantation.

The findings that SUL-121 and SUL-150 significantly improved renal perfusion were further corroborated in isolated porcine intrarenal arteries, where we assessed the effects of SUL-121 and its enantiomers on vasoconstrictions induced by various agonists. SUL-121 and SUL-150, but not SUL-151 inhibited PE mediated contractions, confirming that the vascular effects of SUL-121 are predominately mediated through its *(R)*-enantiomer SUL-150. SUL-121 and SUL-150 enantiomers only had profound effects on α_1_-AR and not on histamine, and U46619 mediated constrictions. Further, none of the metabolites of SUL-150, including Trolox, showed similar effects as SUL-150. As Trolox is a strong antioxidant^21^, these results demonstrate that the effects of SUL-150 are not mediated through scavenging of ROS. In addition, none of the other SUL-compounds investigated (including the enantiomers of SUL-109) inhibited PE-induced contractions. Since SUL-109 has similar effects on mitochondrial function as SUL-121^6^, these findings indicate that the vascular effects of SUL-150 are also not mediated through altered mitochondrial function. The effects of SUL-150 also persisted after removal of the endothelium. Taken together, these results indicate a specific competitive inhibition of α_1_-AR on the vascular smooth muscle by SUL-121 and SUL-150.

To further demonstrate the specificity of SUL-150 for the α_1_-AR we measured calcium signalling in CHO cells overexpressing specific α_1_-AR subtypes. SUL-150 inhibited calcium signalling through α_1_-AR subtypes A and B, whereas the effect on α_1_-AR subtype D was less pronounced. Although the relative expression of α_1_-AR subtypes is unknown in porcine intrarenal arteries, data obtained from rat indicate high mRNA expression levels of α_1A_ and α_1B_ adrenergic receptors in renal blood vessels, whereas α_1D_-AR mRNA was only found in minimal quantities^22^. If these findings may be translated to pig, SUL-121 and SUL-150 therefore antagonize the most predominant α_1_-AR subtypes in porcine intrarenal arteries. Finally, calcium signalling mediated by histamine and U46619 in HeLa cells was unaffected by SUL-150, confirming our findings in renal arteries.

Previously, our group demonstrated that upon stimulation with PE, α_1_-AR transactivate the EGF receptors leading to a stronger contraction of rat aorta^9,10^. Interestingly, the EGFR inhibitor AG1478 caused a competitive inhibition of PE mediated contraction in aorta, similar to the competitive inhibition observed for SUL-150 in porcine intra-renal artery. We therefore investigated the potential involvement of SUL-150 in EGFR transactivation. In porcine intra-renal arteries, AG1478 also caused an inhibition of PE mediated contraction. However, the inhibitory effects of AG1478 were not strictly competitive as no further inhibition could be observed at concentrations higher than 20 µM. Nevertheless, in the presence of a maximal inhibitory concentration of AG1478, SUL150 still caused a further inhibition of PE mediated contraction, indicating that the effects of SUL-150 are independent of EGFRs. The lack of effects of SUL-150 on EGFR was confirmed in HEK293 cells where EGF signalling was found to be unaffected by SUL-150. We therefore conclude that the inhibitory effects of SUL-150 on PE mediated contraction of porcine intra-renal arteries are not mediated through EGFR.

The direct interaction of SUL-150 with the α_1_-AR was demonstrated in a radio ligand binding assay where SUL-150 displaced the α_1_-AR antagonist prazosin at effective concentrations similar as in the vascular and calcium signalling experiments. These findings suggest that SUL-150 and prazosin compete for the same binding site on the α_1_-AR. These findings were further corroborated by our in silico modelling of ligand-receptor interactions. Although our simulation was based on a homology model of the α_1_-AR rather than crystal structure, the estimated free binding energy and involvement of protein residues corresponded with experimentally determined displacement capabilities of SUL-150.

In conclusion, our study demonstrates that antagonism of vascular α_1_-AR can be added to the molecular mechanisms of action of SUL-121 and specifically to its *(R)-*enantiomer SUL-150. This makes SUL-150 unique given that, as of yet, no other SUL compounds demonstrated enantiomer specificity and binding to any vascular receptor. Such new feature is an addition to previously reported effects shared with the rest of the SUL family, including the prevention of ROS formation, maintenance of ATP production and direct ROS scavenging. Interestingly, the criteria proposed for the prevention of ischaemia-reperfusion injury in graft kidneys include the use of vasodilators, antioxidants and anti-inflammatory agents^3^. SUL-150 suits all these criteria and this multi-potency is a distinct advantage over standard antihypertensive agents used to improve graft perfusion and to prevent delayed graft function.

Limitations of the current study include the relatively high concentrations of SUL-150 required to achieve α_1_-AR antagonism. Furthermore, these concentrations are higher than reported for effectively protecting cells against hypothermia^6^. Although we are currently investigating the toxicological parameters of SUL compounds, present data indicate no negative effects of SUL compounds on cell viability at concentrations up to 10 mM^5,6^. Strengths of this study include the use of porcine kidneys which are comparable in size and physiology to human kidneys^23^. As a consequence, our experimental setup is similar in scale to human kidneys, indicating that sufficiently high concentrations of SUL-150 can be reached to improve graft perfusion in clinical machine perfusion setups.

## Methods

### Isolated kidney perfusion

Porcine kidneys from female Dutch Landrace pigs were collected at a local slaughterhouse (Kroon Vlees, Groningen, The Netherlands). Both kidneys were excised together with a section of aorta, ureter and bladder. The aorta was cut open longitudinally and a catheter with a thickened section of silicone was inserted into the renal artery. Using a 50 ml syringe, the kidney was subsequently slowly perfused with 150 ml of cold physiological saline solution supplemented with heparin (5 U/mL). After flushing, 50 ml of cold UW with heparin (5 U/mL) was slowly infused. Subsequently, the same procedure was immediately performed on the contralateral kidney. Next, kidneys were dissected free from aorta and bladder and individually stored in plastic bags on ice for 24 h.

The following day, a single kidney was attached to an in-house developed isolated kidney perfusion system and first flushed with approximately 300 ml of warm saline solution with heparin (5 U/mL). After flushing, kidneys were perfused with warm (37 °C) and oxygenated medium (RPMI 1640) enriched with heparin (5 U/mL) and bovine serum albumin (50 g/L). When medium was detected in the venous effluent of the kidney, perfusion was switched to recirculation with a circulatory volume of approximately 250 ml. The kidneys were allowed to warm by perfusion only, while measuring flow and temperature at a fixed flow pressure of 80 mmHg. Once the renal temperature reached 20 °C, flow was set to constant and the effects of 50 µM SUL-121, SUL-150 and SUL-151 on kidney perfusion pressure were recorded. In separate experiments, pressure was set to a constant (80 mmHg) and flow and temperature were measured before and after addition of 50 µM SUL-150. To minimize the amount of compound needed, the circulatory volume was reduced to 100 ml just before addition of vasoactive compounds to the perfusion medium.

### Tissue preparation and contraction studies in isolated porcine renal artery rings

In an additional series of experiments, the effects of SUL-121, SUL-150 and SUL-151 were assessed in isolated porcine renal artery rings. For this, porcine kidneys were obtained from the local slaughterhouse and transported in normal physiological Krebs buffer (pH 7.4) on ice. The renal artery tree was dissected from the kidney, cleaned of surrounding tissue and cut into equally-sized ring segments (2 mm in length). In some rings, endothelium denudation was performed by gentle rubbing of the intimal surface with a paper clip. Rings were mounted in organ baths for measurement of isotonic displacement as described previously^24^. Arterial rings were washed thoroughly by replacing Krebs buffer and allowed to equilibrate for a period of 60 min under 1.4 g of resting tension before they were assessed for viability by inducing 2 subsequent constrictions with potassium chloride (KCl 60 mM). Rings that failed to produce a threshold displacement of 100 µm were excluded. After washout and stabilization, rings were treated for 30 minutes by incubation with either vehicle (0.1% DMSO), SUL-121, SUL-150 or SUL-151, followed by subsequent stimulation with cumulative doses of phenylephrine (PE, 10 nM – 100 µM). Similarly, following pre-incubation with SUL-150, isotonic constriction to methoxamine (10 nM – 100 µM), the thromboxane A_2_ analogue U46619 (0.3 nM – 10 µM) and histamine (30 nM – 1 mM) was recorded in additional rings. In a separate experiment, the extracellular signal-regulated kinase 1/2 (ERK1/2) pathway was examined by 30 min treatment of rings with the epidermal growth factor receptor (EGFR) inhibitor AG1478 (tyrphostin, N-(3-Chlorophenyl)−6,7-dimethoxy-4-quinazolinamine) and stimulation by phenylephrine. In addition, effects of 30 min pre-incubation with the SUL-121 structural analogues *(R)*-trolox (100 μM), *(S)*-trolox (100 μM), SUL-132 (100 μM) and SUL-138 (100 μM) on constriction responses to PE (10 nM – 100 μM) were assessed.

### Cell culture

CHO-K1 cells were stably transfected with a plasmid containing human α_1_-AR subtypes A, B and D as individual cell lines and grown in 75-cm^2^ cell culture flasks containing Dulbecco’s Modified Eagle’s Medium: Nutrient Mixture F-12 (DMEM/F-12) medium supplemented with 10% FBS, 1% penicillin-streptomycin and 200 µg × mL^−1^ geneticin G418. HeLa cells endogenously expressing histamine and thromboxane (TP) receptors were grown in 75-cm^2^ cell culture flasks containing DMEM/F-12 medium enriched with 10% FBS and 1% penicillin-streptomycin. HEK293 cells were grown in complete DMEM medium enriched with 10% FBS, 1% penicillin-streptomycin and 4.5 g × L^−1^ D-glucose. Flasks were kept in a cell culture incubator at 37 °C in 5% O_2_/95% CO_2_. For calcium assays and radioligand binding, 2 × 10^4^ cells per well were plated 24 hours before measurement on black transparent-bottom treated 96-well plates and clear treated 96-well plates respectively. For ERK1/2 activation assays, 115 × 10^3^ HEK293 cells per well were plated on 6-well plates and serum-starved for 24 hours before they were used in experiments.

### ERK1/2 activation and immunoblotting assays

After 24 hours of serum starvation, triplicate samples of HEK293 cells randomized on 6-well plates were treated with vehicle (0.1% DMSO), SUL-150 (100 µM), or the EGF receptor inhibitor, AG1478 (10 µM), for 30 min and stimulated with recombinant human EGF (1 nM) for 10 min as described previously^9^. Stimulation with EGF was terminated by aspirating the medium, immediately placing 6-well plates on ice and adding 125 µL PBS-based Radioimmunoprecipitation assay buffer (RIPA) lysis buffer containing 1% Nonidet P40, 20 mM sodium orthovanadate and protease inhibitor cocktail. After 15 min incubation, samples were centrifuged at 5000 × g for 5 min at 4 °C, and supernatant collected and stored at −80 °C. Protein concentration was determined using the Bradford protein assay. Phospho-ERK1/2 and GAPDH were detected by SDS-PAGE/immunoblot analysis. Sample proteins were separated with electrophoresis in 12-well Mini-PROTEAN TGX precast 4–20% PAGE gels and transferred onto 0.45μm nitrocellulose membranes. The membranes were immunoblotted by incubation with phosphorylated ERK1/2 (42/44 kDa, pERK (E-4) sc-7383, 1:1000 dilution, Santa Cruz Biotechnology, San Diego, CA) overnight at 4 °C, washed three times for 10 min with Tris-buffered saline and 0.2% Tween 20 and developed using horseradish peroxidase (HRP)-conjugated goat anti-mouse IgG_2b_ antibody (1:1000, sc-2062, Santa Cruz Biotechnology, San Diego, CA) for 2 hours at room temperature. All buffers were enriched with 20 mM NaF to prevent protein dephosphorylation. After subsequent washes, membranes were soaked with Luminol Western Lightning Ultra and band intensities were measured with a GeneGnome (Syngene, Cambridge, UK). To confirm equal loading conditions, expression of GAPDH (37 kDa) was measured using GAPDH primary antibody (Fitzgerald Industries, Acton, MA) and HRP-conjugated goat anti-mouse secondary antibody (1:2000) under conditions described above.

### Intracellular calcium measurements

CHO cells were cultured on a 96 wells plate and loaded with FLIPR Calcium 6 assay kit for 90 min, followed by 30 min incubation with vehicle (0.1% DMSO), SUL-150 (10 μM, 30 μM, 100 μM) and SUL-151 (10 μM, 30 μM, 100 μM) and subsequent stimulation with a single concentration of PE (0.11 nM − 20 μM). Similarly, HeLa cells were treated with SUL-150 (10 μM, 30 μM, 100 μM) and stimulated with histamine (6.27 nM – 10 mM) or U46619 (4.57 × 10^−9^M − 10^−5^M). Wavelengths used for excitation and emission were 485 nm and 525 nm respectively, [Ca^2+^]_i_ was measured in relative fluorescence units (RFU) for 90 seconds in each well and stimulating compounds were added at t = 20 s. Stimulation with agonists and fluorescent measurements were performed in a FlexStation 3 multi-mode microplate reader (Molecular Devices, Wokingham, UK).

### Radioligand binding assay

Intact CHO cells stably overexpressing the α_1A_-AR were cultured to confluence in 96-well plates. Prior to the experiment, cells were washed and the culture medium was replaced by HBSS. Competition curves were built by simultaneous incubation with [7-Methoxy-^3^H]-prazosin (100 nM) and concentrations of SUL-121 enantiomers in the range of 0.5µM-100µM. To calculate specific binding, control reactions were performed using a high dose of unlabelled prazosin (10 µM). Incubations were allowed to proceed for 1 hour at 37 °C. Subsequently, cells were placed on ice, washed three times with ice-cold PBS and lysed by adding 50 µl of 1 N sodium hydroxide (NaOH). Finally, the lysate was transferred to a scintillation vial and counted for 3 minutes.

### Induced fit molecular docking simulation

The primary sequence of human α_1A_-AR was obtained from UniProt database^25^ using reference code P35348 and uploaded to SWISS-MODEL in order to build a homology model^26,27^, resulting in 373 templates. Subsequently, template ligand codes were used to query the PDB database^28^ to obtain structural data in SMILES format, which were processed by Chemmine^29^ to detect similarities with our compound of interest (SUL-121). The SWISS-MODEL template that contained a ligand with the highest similarity score (a D3 dopamine receptor in complex with, Eticlopride, ETQ) was used to align the α_1A_-AR sequence onto a modelled backbone. The resulting homology model was validated by Ramachandran plot^30^ and prepared with Protein Preparation Wizard^31^ by the addition of hydrogens, bond order assignment, generation of partial charges to heteroatoms^32^ and disulphide bonds. Final refinement was performed by hydrogen bond assignment at pH 7.4 and restrained minimization at 0.3 root-mean-square deviation of atomic positions (RMSD).

Prazosin, SUL-150 and SUL-151 structural files were converted from SMILES to 3D structures using LigPrep^31,33^. Protonation states were generated with Epik at pH 7.4 and small molecule energy parameters were computed using the OPLS3 Force Field^34^.

Flexible molecular docking simulation was performed using Induced Fit, a part of the Schrödinger Small-Molecule Drug Discovery suite^35^. A binding centroid was defined between residues involved in antagonist binding confirmed by mutagenesis (PHE312, PHE308 and ASP106) and ligands were docked within 15 Å, using an extended sampling protocol without constrains^12,15^. Residues within 5 Å of resulting ligand poses were refined using Prime to improve ligand conformational sampling^36^. Finally, the Scorpion server was used for the assessment and classification of small molecule-protein interactions^37^ and the final results were rendered using PyMOL 2.0^38^.

### Data and statistical analysis

The data and statistical analysis comply with the recommendations on experimental design and analysis in pharmacology^39^. Values in figures were displayed as mean ± SEM of *n* independent experiments. Vascular constriction responses were expressed as percentage of final response to KCl to account for differences in contractile capability of individual vascular rings. The pEC_50_ values were calculated using a four-parameter logistic regression in Prism 7 (GraphPad Software, Inc., La Jolla, CA) and compared using the Extra sum-of-squares F test with p < 0.05 considered statistically significant. Schild analysis was performed using Gaddum/Schild EC_50_ shift in Prism 7 and pA_2_ values compared using an unpaired parametric two-tailed t-test with Welch’s correction. Fluorescent measurement data was processed in *SoftMax Pro 7* (Molecular Devices LLC, Sunnyvale, CA) and expressed as % of baseline AUC with a 3-fold multiplier using a mean of first 10 measurement points as baseline. Western blot relative protein expression was quantified using Fiji, a distribution of ImageJ^40^.

### Materials

Potassium chloride, DMSO, phenylephrine, methoxamine, histamine, U46619, prazosin, Nonidet P40, sodium orthovanadate and sodium fluoride were purchased from Sigma-Aldrich (Zwijndrecht, Netherlands). SUL-compounds were provided by Sulfateq B.V. (Groningen, Netherlands). Black transparent-bottom treated 96-well plates, clear treated 96-well plates and treated 6-well plates and 75-cm^2^ cell culture flasks were ordered from Corning Inc. (Corning, NY, USA), DMEM/F-12 medium from Lonza (Verviers, Belgium), DMEM medium from Gibco Life Technologies (Paisley, UK), fetal bovine serum from Bovogen Biologicals (Victoria, Australia), penicillin-streptomycin and RPMI Medium 1640 (1X) from Gibco Life Technologies (Grand Island, NY, USA) and Geneticin G418 from; Invitrogen (Carlsbad, CA, USA). FLIPR Calcium 6 assay kit was purchased from Molecular Devices (Wokingham, UK), AG1478 from Focus Biomolecules (Plymouth Meeting, PA, USA), recombinant human EGF from PreproTech (Rocky Hill, NJ, USA), protease inhibitor cocktail from Roche Molecular Diagnostics (Mannheim, Germany), Bradford protein assay, 12-well Mini-PROTEAN TGX precast 4–20% PAGE gels and 0.45 μm nitrocellulose membranes from Bio-Rad (Hercules, CA, USA), Tween 20 from Promega (Madison, WI, USA), heparin from Leo Pharma BV (Amsterdam, The Netherlands), bovine serum albumin from Amresco, LLC (Solon, OH, USA) and Luminol Western Lightning Ultra and [7-Methoxy-^3^H]-prazosin from PerkinElmer (Waltham, MA, USA).

## Electronic supplementary material

Supplementary information
