# Supplementary material for: The (R)-enantiomer of the 6-chromanol derivate SUL-121 improves renal graft perfusion via antagonism of the α1-adrenoceptor
Source: Sci Rep. 2019 Jan 9;9:13. doi: 10.1038/s41598-018-36788-0 (PMC6327096; doi:10.1038/s41598-018-36788-0)
Supplement: Supplementary file 1 — Supplementary information [file 41598_2018_36788_MOESM1_ESM.pdf]

The *(R)*-enantiomer of the 6-chromanol derivate SUL-121 improves renal graft perfusion via antagonism of the α_1-adrenoceptor

D Nakladal[1,2], H Buikema[1], A Reyes Romero[3], S. P. H. Lambooy[1], J Bouma[1], G Krenning[4,5], P Vogelaar[5], A C van der Graaf[5], M R Groves[3], J Kyselovic[6], R H Henning[1] and L E Deelman[1]

1. Department of Clinical Pharmacy and Pharmacology, University of Groningen, University Medical Center Groningen, Hanzeplein 1, 9713GZ Groningen, the Netherlands.
2. Department of Pharmacology & Toxicology, Faculty of Pharmacy, Comenius University in Bratislava, Odbojárov 10, 832 32, Bratislava, Slovakia
3. Department of Drug Design, School of Pharmacy, University of Groningen, Antonius Deusinglaan 1 Postbus 196, 9700 AD, Groningen, The Netherlands.
4. Cardiovascular Regenerative Medicine, Dept. Pathology and Medical Biology, University of Groningen, University Medical Center Groningen, Groningen, The Netherlands
5. Sulfateq B.V., Admiraal de Ruyterlaan 5, 9726GN, Groningen, The Netherlands
6. 5th Department of Internal Medicine, Faculty of Medicine, University Hospital, Commenius University, Bratislava, Slovak Republic

Supplementary figures

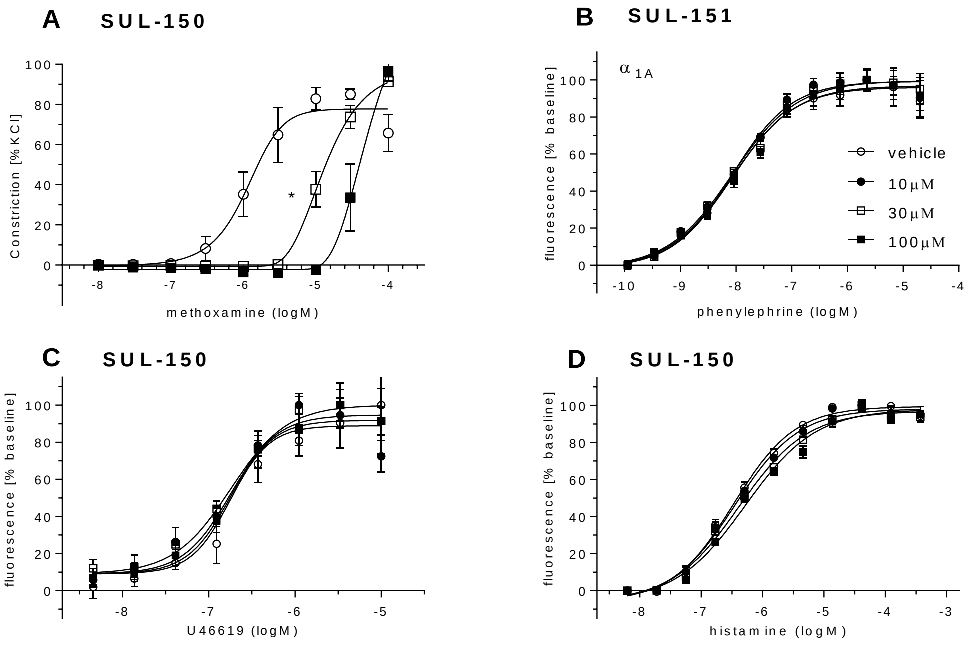

Supplementary Figure 1. (A) Effect of SUL-150 on methoxamine-induced constriction in endothelium-denuded PIRA, (B) effect of SUL-151 on PE-induced calcium transients in α_{1A}-AR transgenic CHO cells, (C) U46619 and (D) histamine-induced calcium influx in HeLa cells after pre-treatment with SUL-150. Data from 2-3 experiments (n=4-6 per group).

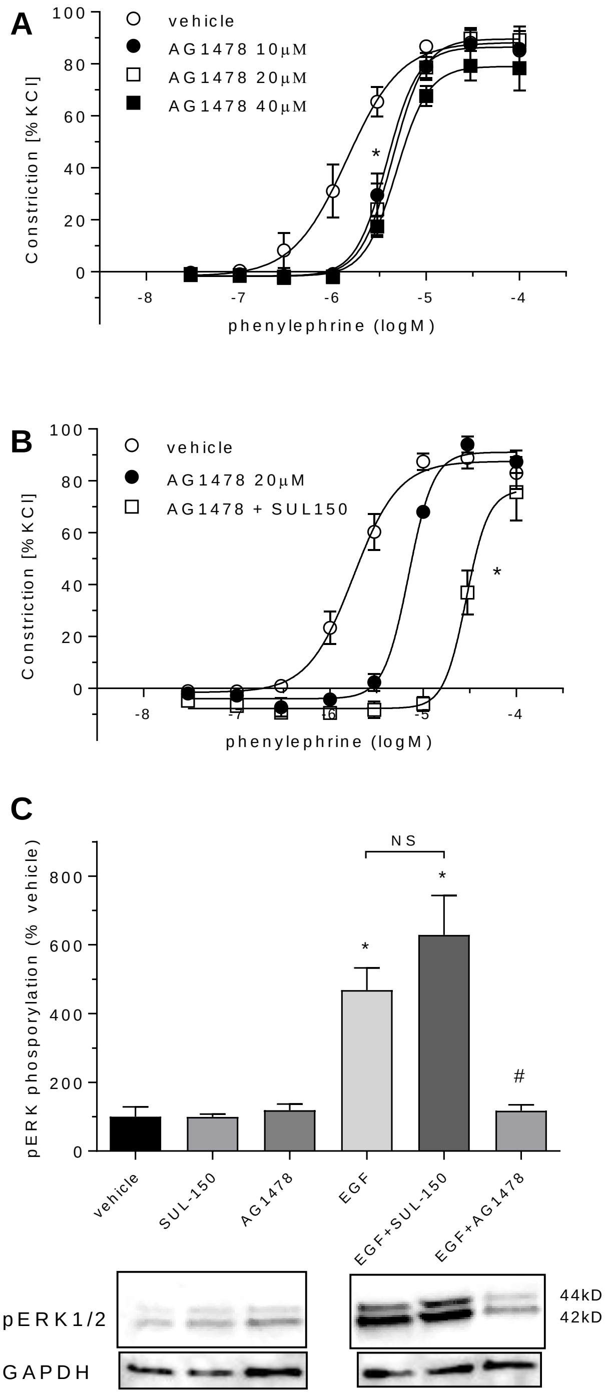
A
vehicle
AG1478 10µM
AG1478 20µM
AG1478 40µM
Constriction [%KCl]
100
80
60
40
20
0
-8
-7
-6
-5
-4
phenylephrine (logM)
*
B
vehicle
AG1478 20µM
AG1478 + SUL150
Constriction [%KCl]
100
80
60
40
20
0
-8
-7
-6
-5
-4
phenylephrine (logM)
*
C
pERK phosporylation (% vehicle)
800
600
400
200
0
NS
*
*
#
vehicle
SUL-150
AG1478
EGF
EGF+SUL-150
EGF+AG1478
pERK1/2
GAPDH
44kD
42kD

Supplementary Figure 2. The inhibitory action of SUL-150 on phenylephrine-induced contractions in porcine intrarenal artery rings is not dependent on EGFR transactivation. (A) Porcine intrarenal arteries were pre-treated with the EGFR inhibitor AG1478. Increasing concentrations of AG1478 (10, 20 and 40µM) all caused a similar maximal inhibition of PE induced contractions. (B) In the presence of 20µM AG1478, SUL-150 still caused a further inhibition of PE induced contractions. Therefore, despite maximal inhibition of EGFR transactivation by AG1478, SUL-150 still demonstrated an additional inhibitory effect on PE-mediated constriction * $p < 0.05$ AG1478 + SUL-150 vs AG1478. Data from 2-3 experiments (n=4-6 per group). (C) EGF signalling was further studied by measuring EGF mediated activation of pERK1/2 in HEK293 cells using Western blotting. Stimulation of HEK293 cells with EGF resulted in a substantial phosphorylation of ERK1/2, which was not influenced by SUL-150, while this effect was effectively inhibited by AG1478. These experiments therefore demonstrate lack of effects of SUL-150 on EGF signalling. Representative cropped blots for pERK1/2 and GAPDH as loading control and are shown beneath the figure. Groups in the blots are respective to the bar graph. EGF (1nM) caused a significant increase in ERK1/2 phosphorylation which was unaffected by SUL-150 (50µM). AG1478 (10µM) fully blocked ERK1/2 phosphorylation by EGF. * $p < 0.05$ vs vehicle, # $p < 0.05$ vs EGF, n=3 per group.

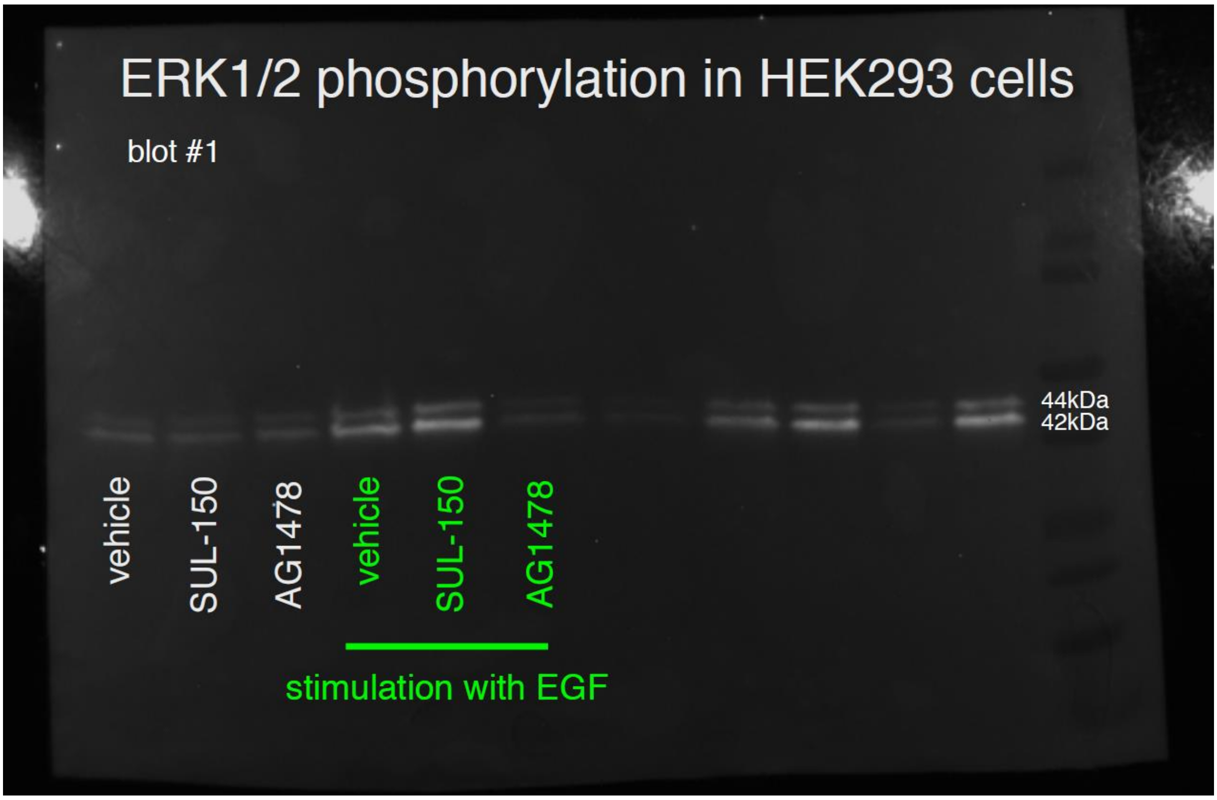

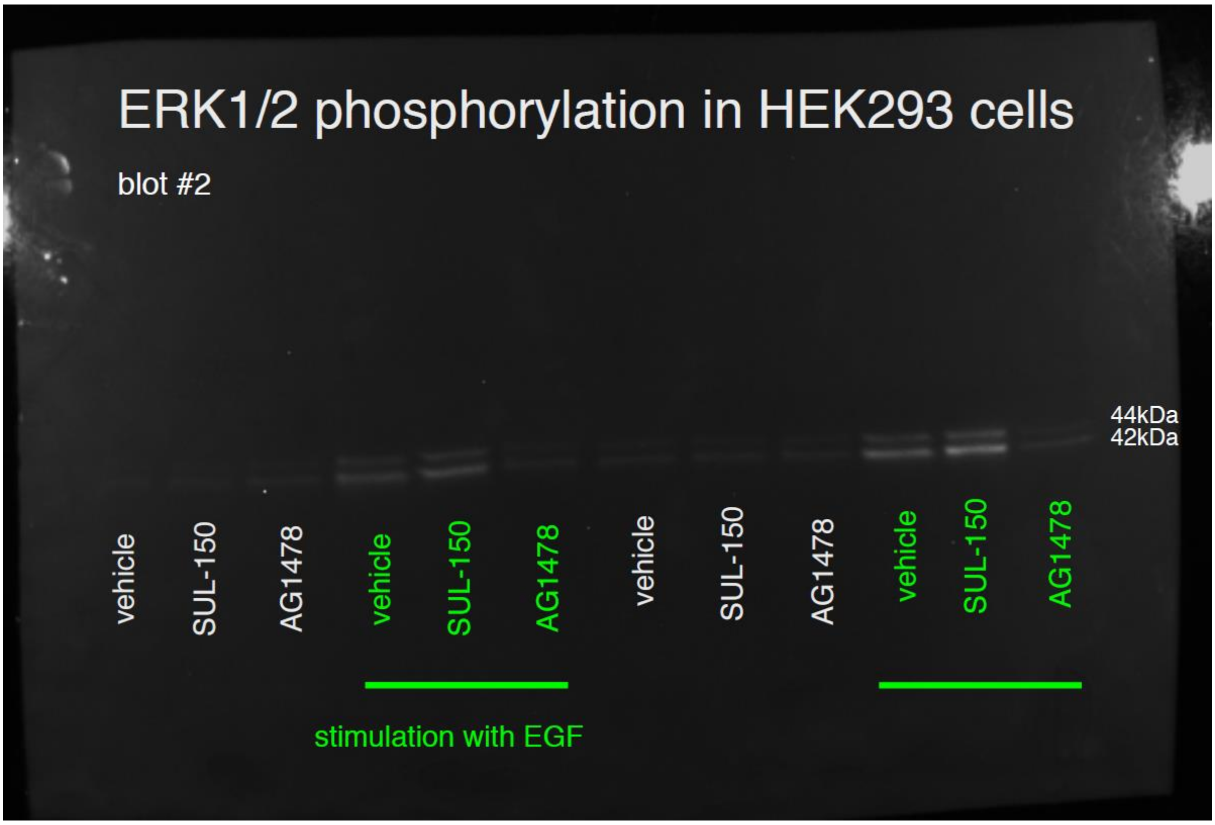

Supplementary Figure 3. Western blots for pERK1/2 measured in HEK293 cells after pre-incubation with vehicle, the EGF receptor inhibitor AG1478 or SUL-150 and stimulation with vehicle or EGF.

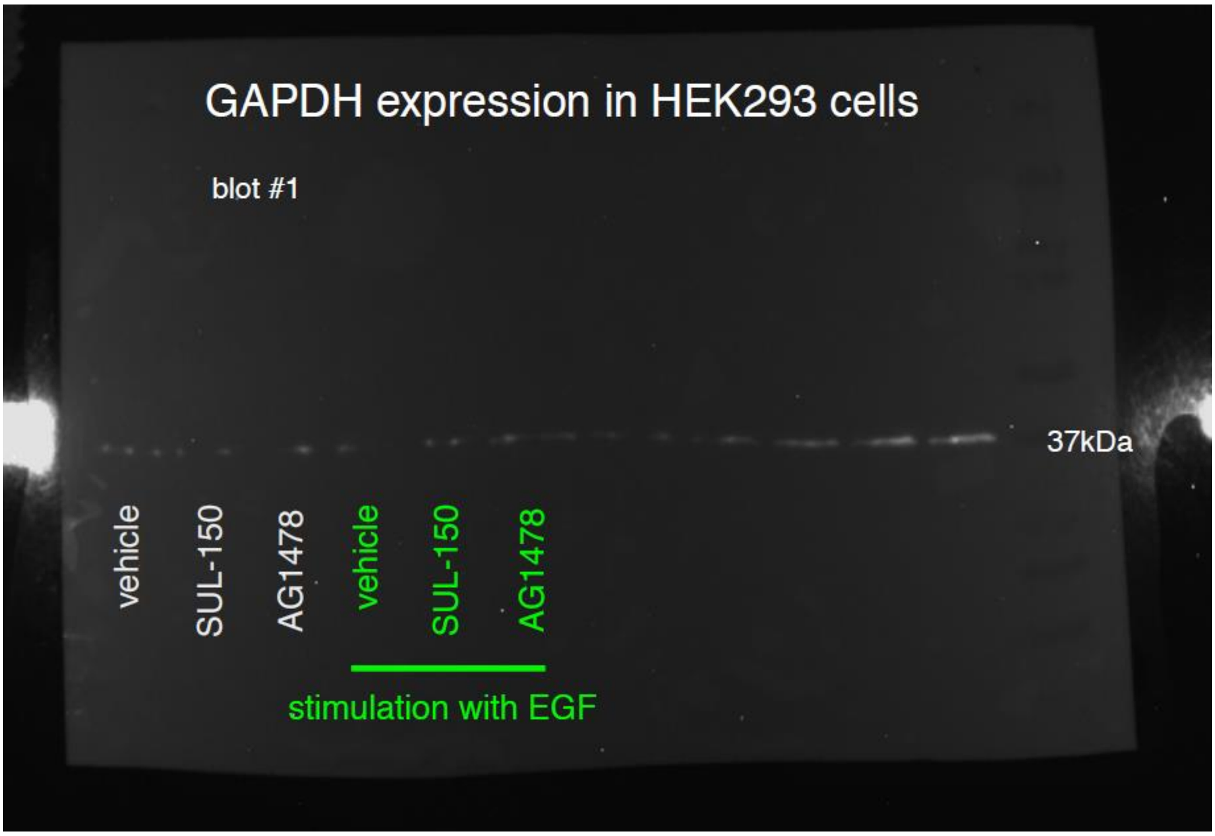

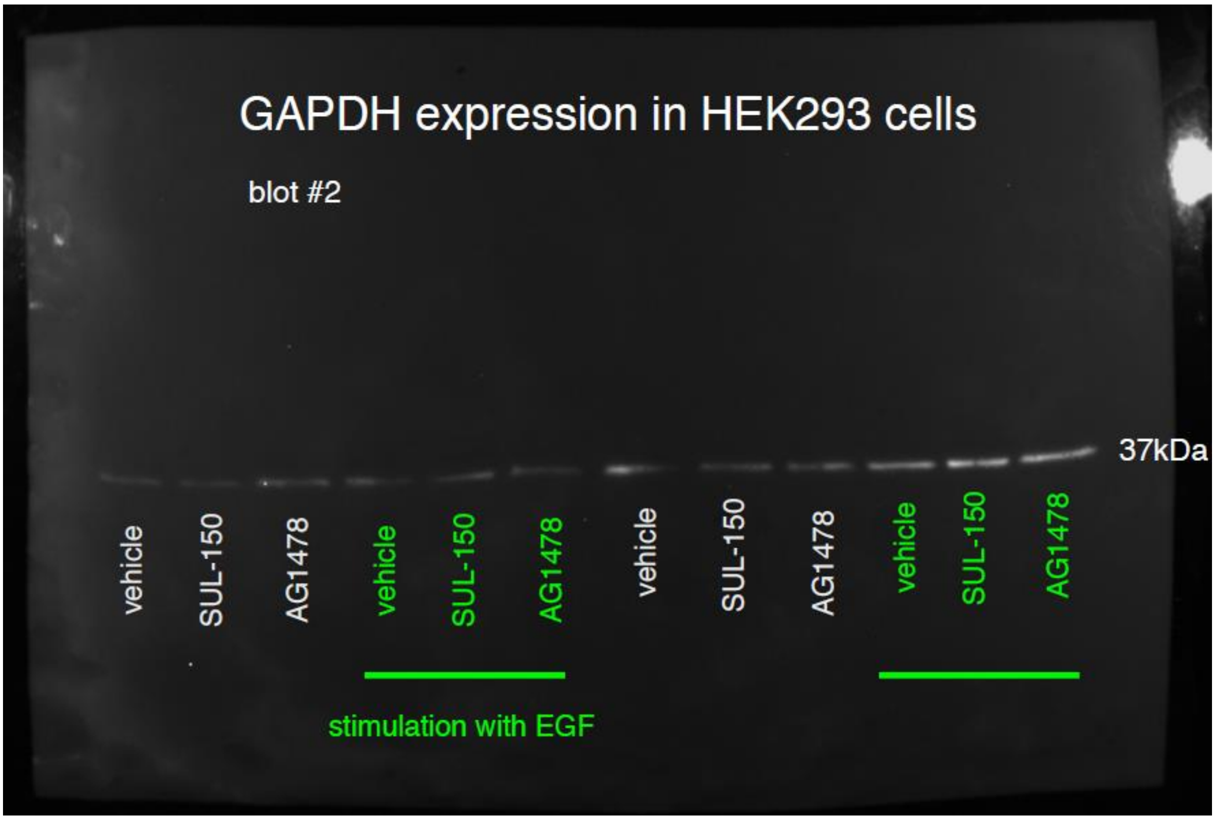

Supplementary Figure 4. Western blots for GAPDH as loading control measured in HEK293 cells after pre-incubation with vehicle, the EGF receptor inhibitor AG1478 or SUL-150 and stimulation with vehicle or EGF.

Supplementary tables

Supplementary Table 1. The effect of SUL-compounds on E_{max} values for various agonists in porcine intrarenal arteries and in vitro cell models

compound	*agonist*	*model*	*vehicle*	*concentration*		
				10µM	*30µM*	*100µM*
SUL-121	PE	PIRA	95.6±1.7	103.0±2.0*	105.0±2.6	107.0±4.5
SUL-150	PE	PIRA	93.0±2.9	101.0±6.0	89.0±12.4	97.9±6.2
		CHO + α_{1A}	98.7±0.9	100.4±1.1	99.0±1.4	99.1±1.6
		CHO + α_{1B}	98.7±2.1	99.9±2.5	98.5±2.9	101.6±4.5
		CHO + α_{1D}	94.7±3.3	101.1±3.7	101.2±4.3	97.2±4.8
	methoxamine	PIRA	82.7±3.5		85.7±3.4	84.1±6.8
		PIRA[a]	77.8±0.1		97.2±0.2	96.5±4.9
	histamine	PIRA	88.3±3.0		92.2±3.7	101.0±4.0
		HeLa cells	99.4±1.4	98.0±1.5	96.9±1.6	97.9±1.7
	U46619	PIRA	82.8±1.6		80.4±1.8	90.1±1.8*
		HeLa cells	94.4±6.5	89.7±5.6	98.4±6.4	95.1±6.1
SUL-151	PE	PIRA	93.0±3.7	102.0±5.4	80.3±3.7	98.2±3.3
		CHO + α_{1A}	95.4±2.5	98.5±2.5	99.8±2.6	98.1±2.7

Each value represents the mean ± standard error of the mean (SEM). (n=4-6 per group). porcine intrarenal artery (PIRA), a: endothelium-denuded arterial rings, * $p < 0.05$ vs vehicle

Supplementary Table 2. Effects of SUL-150 on pEC_{50} values for methoxamine, histamine and U46619 mediated constrictions in porcine intrarenal arteries.

		concentration of SUL-150	
agonist	*vehicle*	*30µM*	*100µM*
methoxamine	5.73±0.06	4.73±0.03*	4.32±0.03*
histamine	4.94±0.03	4.86±0.08	4.85±0.10
U46619	7.65±0.05	7.58±0.06	7.50±0.04*

pEC_{50} values are –log transformed. Each value represents the mean ± standard error of the mean (SEM). (n=4-6 per group). * $P<0.05$ vs vehicle.
